# Retraction Note: CircPSMC3 suppresses the proliferation and metastasis of gastric cancer by acting as a competitive endogenous RNA through sponging miR-296-5p

**DOI:** 10.1186/s12943-023-01718-w

**Published:** 2023-01-16

**Authors:** Dawei Rong, Chen Lu, Betty Zhang, Kai Fu, Shuli Zhao, Weiwei Tang, Hongyong Cao

**Affiliations:** 1 Department of General Surgery, Nanjing First Hospital, Nanjing Medical University, Nanjing, Jiangsu China; 2 Michael G. DeGroote School of Medicine, McMaster University, Hamilton, Ontario Canada; 3 Department of General Clinical Research Center, Nanjing First Hospital, Nanjing Medical University, Nanjing, Jiangsu China

**Retraction Note: Mol Cancer 18, 25 (2019)**

**https://doi.org/10.1186/s12943-019-0958-6**

The Editor-in-Chief has retracted this article at the authors' request. After publications, concerns were raised regarding image overlap in Figs. 4 and 5, which the authors addressed via a Correction [1]. However, further concerns have been raised following the correction regarding breaks and potential overlaps in Figs. 4C, 4G, 5C and 5H. In the revised Fig. 5H, there appears to be overlap between the images representing MGC803 and AGS cells in both treatment groups.

Furthermore, BGC823, MGC803 and SGC7901 cells used in this study are known to be contaminated (or partially contaminated) with HeLa cells, making them an unsuitable model for gastric cancer [2].

The authors have provided the raw data to address the image concerns, but have requested to retract the article due to the cell line contamination.

Shuli Zhao, Weiwei Tang and Hongyong Cao agree to this retraction. Chen Lu and Kai Fu have not responded to any correspondence from the editor or publisher about this retraction. The Publisher has not been able to obtain current email addresses for Dawei Rong and Betty Zhang.
